# Endometriosis specific vaginal microbiota links to urine and serum *N*-glycome

**DOI:** 10.1038/s41598-024-76125-2

**Published:** 2024-10-25

**Authors:** John MacSharry, Zsuzsanna Kovács, Yongjing Xie, Barbara Adamczyk, Caitriona Walsh, Fiona Reidy, Fionnuala M. McAuliffe, Mark T Kilbane, Patrick J Twomey, Pauline M. Rudd, Mary Wingfield, Michael Butler, Douwe van Sinderen, Louise Glover, Radka Saldova

**Affiliations:** 1 School of Microbiology, University College Cork, Cork, Ireland; 2 APC Microbiome Ireland, University College Cork, Cork, Ireland; 3 National Institute for Bioprocessing Research and Training (NIBRT), Belfield, Blackrock, Co. Dublin Ireland; 4 Merrion Fertility Clinic and National Maternity Hospital, Dublin, Ireland; 5 UCD Perinatal Research Centre, University College Dublin, National Maternity Hospital, Dublin, Ireland; 6 Department of Clinical Chemistry, St. Vincent’s University Hospital, Dublin, Ireland; 7 UCD School of Medicine and Medical Science, University College Dublin, Dublin, Ireland; 8 UCD School of Medicine, College of Health and Agricultural Science (CHAS), University College Dublin, Dublin, Ireland

**Keywords:** Endometriosis, Microbiota, *N*-glycans, Urine, Serum, Biomarkers, Glycobiology, Reproductive disorders, Biomarkers, Clinical microbiology

## Abstract

Endometriosis is a chronic systemic disease, which results in endometrial-type tissue growing outside the uterus, and affects approximately 10% of reproductive-aged women worldwide. Its aetiology is poorly understood, and there is currently no long-term cure. Development and persistence of the disease depend on several coexisting factors including the vaginal microbiome. However, the role played by this important entity in endometriosis and its systemic involvement is not fully understood. Here, we investigated the vaginal microbiota, the serum and urine glycome, and antibody glycosylation in endometriosis patients. We reveal an endometriosis-specific vaginal microbiota in patients, being distinct from that present in a control group. Endometriosis patients were typified by a loss of the dominant *Lactobacillus* species, i.e. *Lactobacillus iners*, increased bacterial diversity and the presence of species such as *Anaerococcus senegalensis*,* Prevotella jejuni*,* Porphyromonas bennonis* and *Anaerococcus octavius*. The presence of trigalactosylated and triantennary serum glycans and urine core fucosylated mono-antennary glycans from IgG correlated with the vaginal presence of the bacterium *A. senegalensis* in endometriosis patients. Urine glycans did not differ in endometriosis, but urine IgG identified four novel sulfated glycans differing from serum IgG indicating functional relevance. Our findings contribute to understanding the relationships between the vaginal microbiota and the serum and urine glycome on the one hand, and endometriosis on the other. Further functional studies are warranted.

**Supplementary Information:**

The online version contains supplementary material available at 10.1038/s41598-024-76125-2.

## Introduction

Endometriosis is a chronic gynaecological disease in which endometrial-like tissue grows outside the uterine cavity^1^. Affecting approximately 10% of reproductive-aged women, it is a major cause of infertility as well as causing significant dysmenorrhoea and pelvic pain, while also being associated with increased risks of cancer and autoimmune diseases^1^. The clinical presentations vary and it may also manifest outside the female reproductive tract rendering it a systemic disease^2^. It affects metabolism in liver and adipose tissue, causing systemic inflammation, and alters gene expression in the brain resulting in pain^2^. The aetiology of endometriosis is poorly understood, and laparoscopy, an invasive procedure, is the gold standard for diagnosis^1^. Recently, a promising novel, non-invasive diagnostic approach has emerged using a salivary miRNA signature^3^, pointing to the systemic mucosal nature of the condition. Treatment may involve surgical intervention or medical and hormone therapy, although neither treatment is curative and thus endometriosis is considered a chronic disease with high rates of recurrence throughout reproductive life^1^. The development and persistence of this disease depend on several coexisting factors, including genetic predisposition, prenatal exposure to endocrine-disrupting chemicals, the vaginal microbiome, the immune system and sex hormones^1^.

The importance of the vaginal microbiome in endometriosis is being increasingly recognised. One proposed mechanism of endometriosis pathogenesis (bacterial contamination hypothesis) involves microbial stimulation of pathogen recognition receptors, which causes activation of pro-inflammatory pathways and innate immune cell infiltration, in turn resulting in the clinical symptoms of endometriosis^4,5^. Cervicovaginal bacteria have been found to be a major modulator of host inflammatory responses in the female genital tract inducing pro-inflammatory cytokines via the TLR4 and NF-κB signalling pathway^6^. Inflammatory cytokines along with other factors in peritoneal fluid from endometriosis patients were found to be embryotoxic^7^ and may contribute to endometriosis-associated infertility^8^. Increasing evidence has shown that the endometrial microbiota is important for successful implantation, i.e. endometria lacking a *Lactobacillus*-dominant microbiota are observed to have reduced successful egg/embryo implantation, pregnancy, ongoing pregnancy and live birth^8^. Endometriosis has been found to be associated with increased *Proteobacteria*, *Enterobacteriaceae*,* Streptococcus* spp. and *Escherichia coli* across various tissue types, including the gut, vagina, cervix, endometrium, fallopian tubes, ovaries, peritoneum, peritoneal fluid, follicular fluid, menstrual blood and ectopic endometriosis lesions, while particular species belonging to the genus *Lactobacillus*, which represent common resident microbes of the vagina, have been found to be decreased, with also increasing evidence suggesting intracellular microbial infection as a risk factor^1,9–12^. Additionally, vaginal, cervical and endometrial microbiota compositional information can be used to predict infertility due to endometriosis, indicating the significance of the microbiota in endometriosis-related infertility^13^.

Carbohydrates, also known as ‘glycans’, are important for signal transduction and metabolic processes. They influence the properties of proteins and determine immunological responses, with altered glycosylation patterns being associated with various diseases, including chronic inflammatory conditions and cancer^14^. Therefore, glycan profiles characteristic for disease have promising potential as both clinical markers and therapeutic targets in endometriosis^1^. Changes in glycosylation have been reported in endometriosis such as altered glycosylation of plasma glycoproteins in secretory phase endometrial tissues from women with advanced endometriosis compared to controls, and aberrant glycosylation and expression of endometriosis-associated peritoneal haptoglobin, alterations in sialylation on endometrial cells and altered glycosylation on all serum glycoproteins and serum IgG of endometriosis patients^1,15–17^. Changes in serum/plasma *N*-glycosylation occur mainly in acute phase proteins and IgG^14^. Glycosylation patterns in the urine of endometriosis patients have not been studied to date, in spite of the fact that urine can be obtained by non-invasive means, is less complex, and is more stable than blood-derived samples^18^. The urinary proteome consists of proteins from the glomerular filtrate of plasma and the proteome of the urogenital tract^19^. Therefore, the urinary proteome and/or glycome may reflect both systemic and urogenital physiological conditions. Despite these advantages, urinary glycan biomarker research is only recently evolving due to the very low protein concentration normally present in urine and the presence of interfering compounds^19^. To date, no studies have reported a comprehensive view of overall urinary protein or IgG *N*-glycosylation profiles in endometriosis.

Possible correlations between the glycome and microbiota in endometriosis have not been reported yet, however, there is evidence that blood group antigens and other glycans expressed on mucosal surfaces can serve as receptors for microorganisms, parasites and viruses having a role in host susceptibility to infections^20,21^. Therefore, associative studies to link the glycome profiles and microbiome composition may provide insights into the possibility that glycans play an important role in susceptibility to endometriosis.

In this study, we investigated the microbiota and *N*-glycome from non-invasively collected samples of high vaginal swabs, serum and urine, respectively, from women with endometriosis and women with similar complaints but no disease. We explored the interconnectivity of microbiota, glycome and clinical features. Our results revealed novel microbes associated with endometriosis as well as complex relationships, not only showing promise for improved non-invasive diagnosis, but also facilitating further insight into this enigmatic systemic disease.

## Results

### The vaginal microbiota of endometriosis

**Fig. 1:**
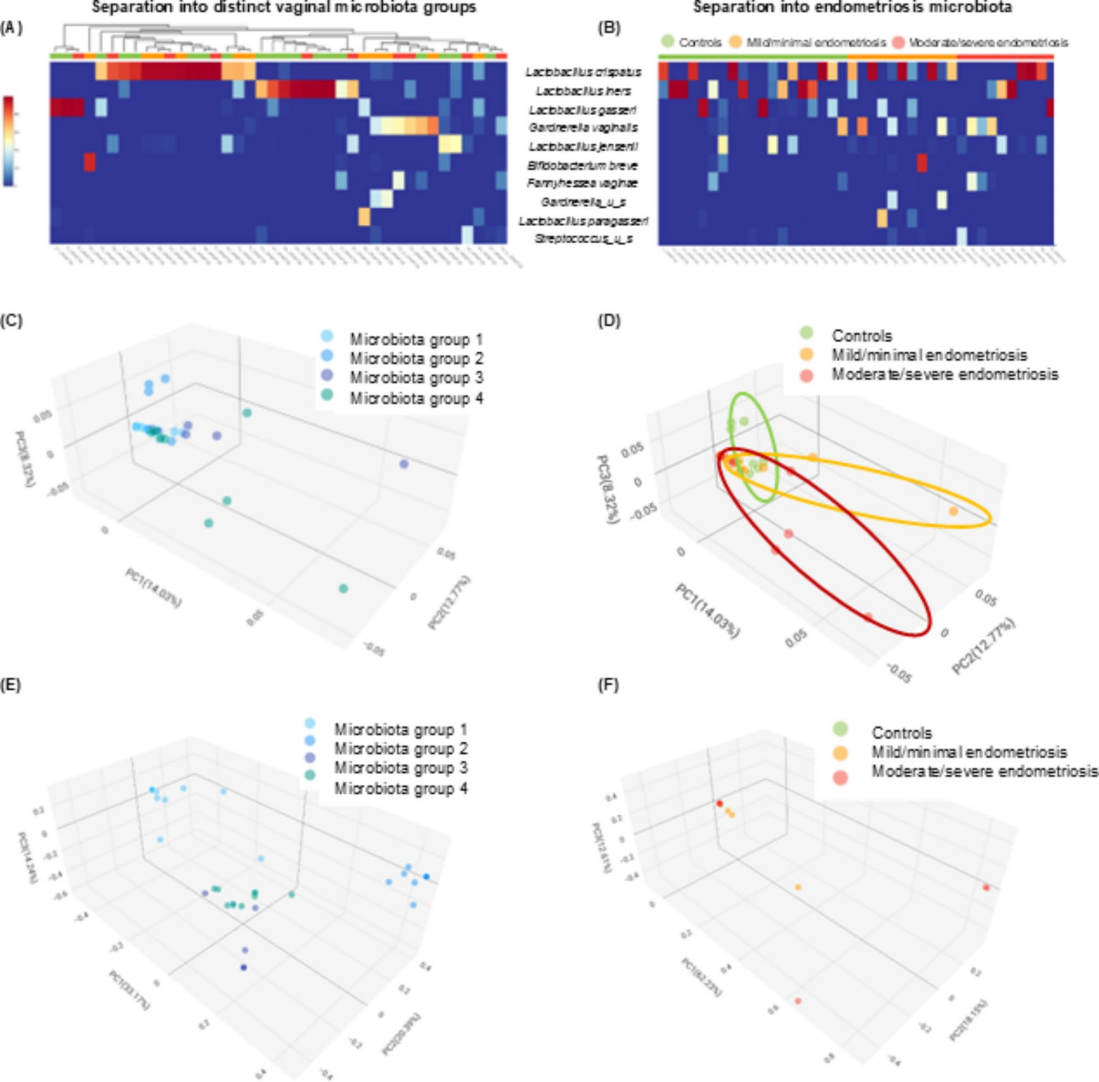
Shotgun metagenomic analysis of vaginal swabs reveal distinct microbiota cohorts in endometriosis samples (*n* = 40). (**A**) Distinct vaginal microbiota groups were identified, sample clustering heatmap of most dominant species (top 10) revealed distinct cohorts in the endometrial vaginal swabs. Group1- *Lactobacillus crispatus*, Group2-*Lactobacillus iners*, Group3-*Lactobacillus gasseri*, and Group4- *Gardnerella vaginalis* dominant. (**B**) Heatmap of dominant species based on clinical scoring. (**C**) PCA of samples based on bacteria reveal distinct microbiota groups. (**D**) PCA of samples based on bacteria respective to clinical scoring revealed distinct groups in the endometrial vaginal swabs. (**E**) PCOA beta diversity Bray Curtis separation based on bacteria of microbiome groups. (**F**) PCOA beta diversity Bray Curtis separation based on fungi of clinical cohorts (more details about beta diversity in clinical cohorts are in Table S1B).

**Fig. 2:**
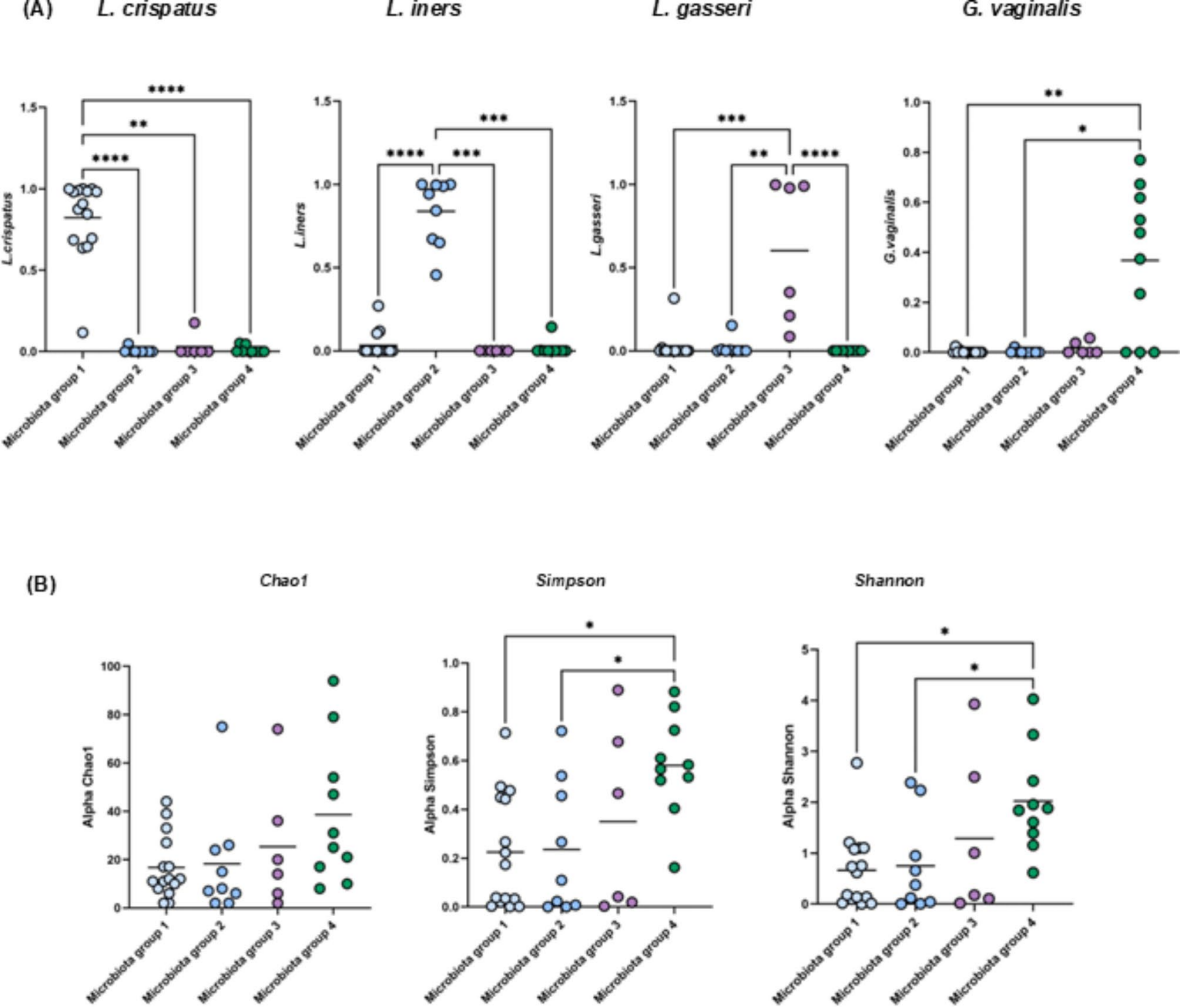
Microbiota groups specific species and alpha diversity. (**A**) Abundance of four most abundant bacteria in four microbiota cohorts according to their dominant species and (**B**) alpha diversity based on bacteria in each group.

Shotgun metagenomic sequencing (WGS) of high vaginal swabs (Table S1A) revealed that the patient cohort (Table 1) clustered based on the most domains microbiota into four distinct groups: subjects with a vaginal microbiota dominated by *Lactobacillus crispatus* (group 1), *Lactobacillus iners* (group 2), *Lactobacillus gasseri* (group 3), or *Gardnerella vaginalis* or other dominant microbes (group 4) with endometriosis clinical scoring cohorts spanning across these groups (Figs. 1A and 2A, Fig. S1). These microbiota groups do not clearly separate using PCA, however there is a more distinct bacterial composition for some of the patients corresponding to groups 3 and 4 (Fig. 1C). These microbiota groups also display substantial beta diversity, with all groups significantly separate from each other (*p* = 0.001, PCOA Bray-Curtis based on bacteria) (Fig. 1E). The *Gardnerella vaginalis*-dominant group 4 was shown to display the highest alpha diversity levels (Fig. 2B).

**Table 1:** Clinical characteristics of endometriosis patients and matched controls.

Summary of clinical characteristics	Controls (19)		Mild/minimal endometriosis (11)		Moderate/severe endometriosis (10)	
Age	38, 35–40		35, 33–37		33, 30–35	
BMI	23.0, 21.1–28.0		23.3, 22.6–24.1		25.6, 22.2–26.8	
Antibiotics	4		0		2	
Insulin (mU/L)	6.2, 5.4–7.9		5.5, 2.7–7.7		8.9, 4.4–11.9	
Fasting glucose(mmol/L)	4.7, 4.4–5.2		4.7, 4.5-6.0		5.4, 4.5–5.6	
Hb1A1c (mmol/mol)	32.0, 30.5–35.0		34.0, 30.5–35.3		32.0, 31.0–33.0	
Inflammatory gynaecological conditions	6		1		0	
Endometrioma	0		0		8	
Peritoneal endometriosis	0		8		7	
Cycle length	28, 27–30		28, 26–28		30, 28–34	
Phase of the cycle (^#^sample numbers)	Follicular (7)	Luteal (10)	Follicular (4)	Luteal (7)	Follicular (6)	Luteal (4)
LH (IU/L)	9.6, 8.7–12.0	4.5, 2.8–6.6	5.3, 3.0-5.9	7.6, 2.4–13.0	9.3, 0.8–10.9	5.2, 4.3–7.5
FSH (U/L)	6.6, 6.1–10.0	3.0, 2.6–3.2	6.7, 4.2–8.9	3.6, 3.1–5.5	6.2, 5.5–6.9	7.3, 2.4–12.0
Oestradiol (pmol/L)	297.4, 145.0-347.4	559.3, 538.6-712.3	*413.5	686.7, 438.9-881.5	347.6, 263.5-444.9	187.6, 123.3-251.8
Progesterone (nmol/L)	2.1, 0.7–3.6	42.3, 35.3–45.6	1.0, 0.8-1.0	30.3, 15.4–48.2	0.8, 0.5–2.7	19.1, 12.8–27.1
Oestradiol/progesterone ratio	166.2, 87.7-278.1	13.0, 11.7–23.5	*751.8	22.1, 13.9–274.0	130.2, 101.7–295.0	59.3, 37.1–81.5

^#^Two samples have unknown phase of the cycle (controls), *only one data value available.

**Fig. 3:**
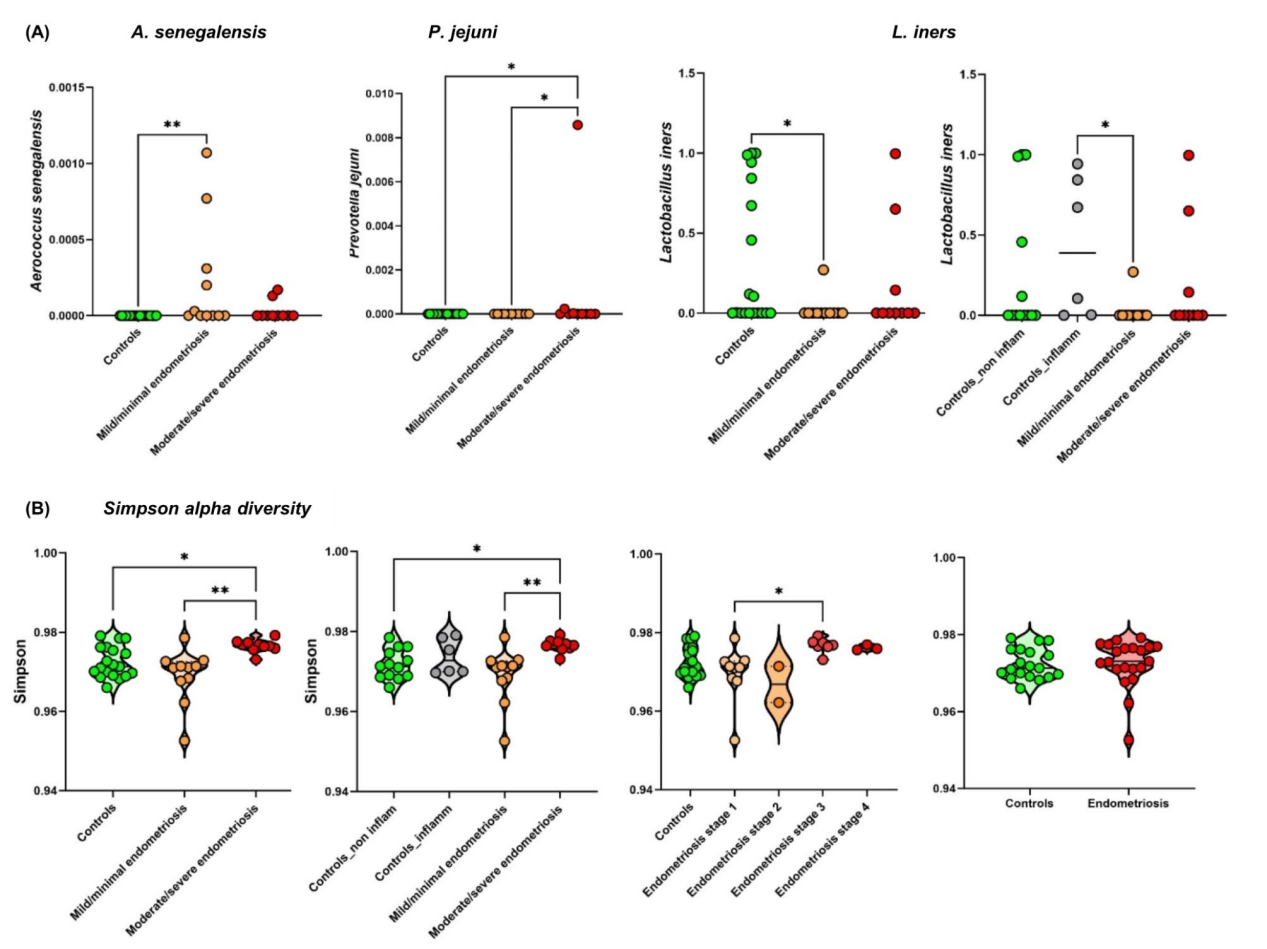
Clinical cohorts specific species and alpha diversity. (**A**) Specific bacteria for endometriosis and (**B**) associated alpha diversity Simpson based on GO.

**Fig. 4:**
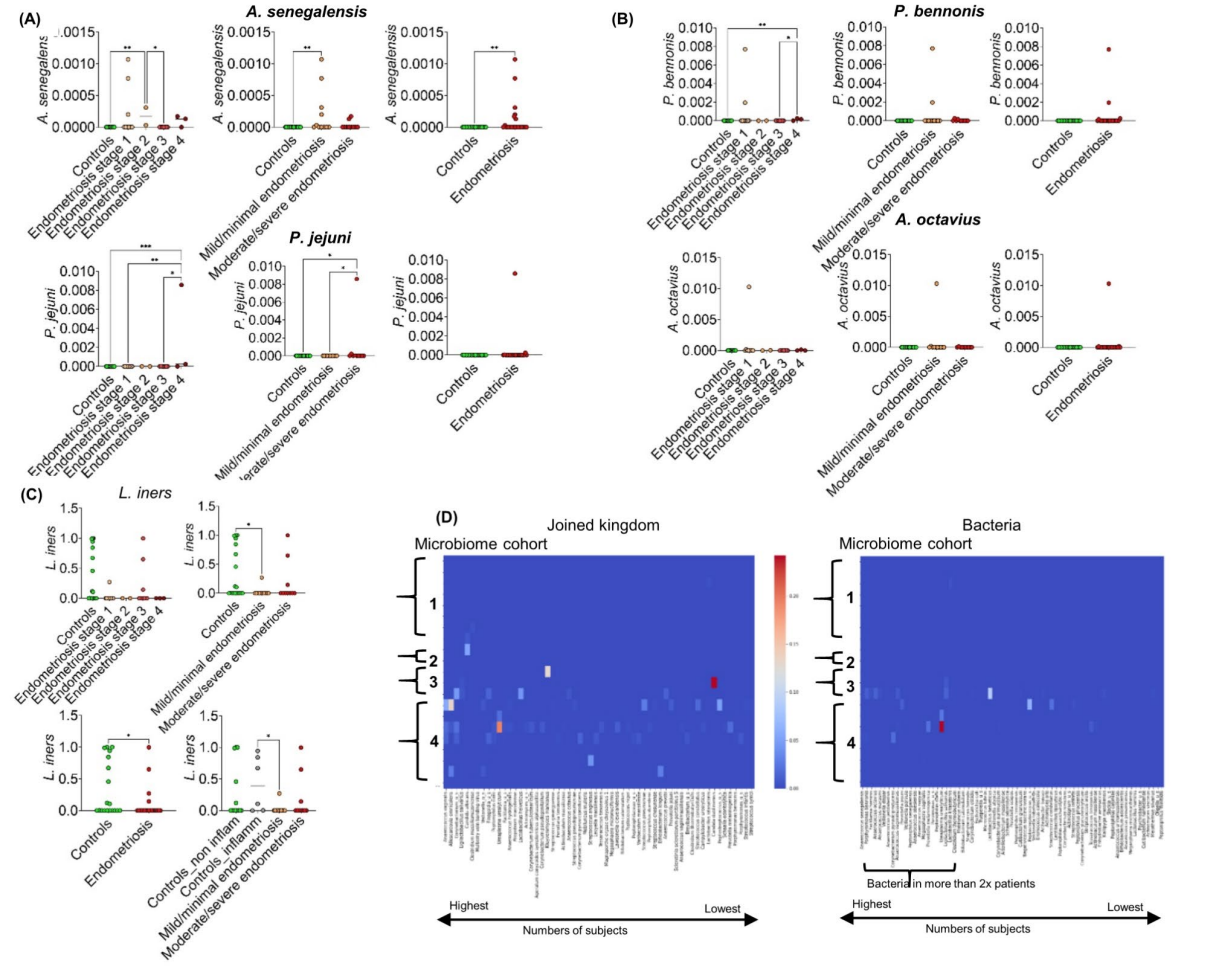
Specific bacteria for endometriosis, separation into clinical stages. (**A**) *Anaerococcus senegalensis* and *Prevotella jejuni*. (**B**) *Porphyromonas bennonia* and *Anaerococcus octavius*. (**C**) *Lactobacillus iners*. (**D**) Heat maps of microbes (joined kingdom) and bacteria found only in endometriosis patients; the relative abundance values are from Table S1A and microbes with their numbers are summarized in Table S1C.

Clinical cohorts did not clearly separate by dominant microbiota (Fig. 1B), however, they did cluster together on PCA, based on total microbiota composition (Fig. 1D). The dominant microbial species were not distinct between the clinical cohorts, although microbiota group 2 corresponds to the smallest number of endometriosis cases (Figure S1). Many samples from endometriosis patients, especially corresponding to microbiome groups 3 and 4, were enriched for specific low abundant bacteria, namely in *Porphyromonas bennonis*, *Anaerococcus octavius* and *Anaerococcus senegalensis* predominantly in mild/minimal endometriosis with *Prevotella jejuni* in moderate/severe endometriosis (Figs. 3A and 4A and B, Table S1B, C). Specific microbes from joined kingdom and bacteria for controls and endometriosis patients separately were plotted in heatmaps (Fig. 4D) and patient numbers were assessed (Supplementary Table S1C). Endometriosis patients were enriched, with more than three patients positive for, in low abundant bacteria such as *Anaerococcus vaginalis*,* Alloscardovia omnicolens*,* Corynebacterium_u_s* (joined kingdom), *Anaerococcus senegalensis*,* Porphyromonas bennonis*,* Facklamia hominis*,* Anaerococcus octavius*,* Anaerococcus marasmi*,* Veillonella dispar*,* Anaerococcus jeddahensis*,* Corynebacterium pyruviciproducens*,* Anaerococcus vaginimassiliensis*,* Prevotella jejuni* and *Veillonella parvula* (bacteria) (Table S1C). Interestingly, these apparent endometriosis-specific bacteria were mainly found in microbiome groups 3 and 4 which also displayed the highest alpha diversity (Fig. 2B, Fig. 4D).

Controls were sub-divided based on whether they had other gynaecologic inflammatory conditions (hydrosalpinx, salpingitis, sactosalpinx) to see if there is a specific signature associated with such inflammatory conditions and how these subgroups separate from endometriosis patients. *Lactobacilus iners* came as most significantly altered bacteria in this control cohort (Table S1B). Endometriosis was associated with a decrease in the presence of *Lactobacillus iners* compared to these controls (Table S1B, Figs. 3A and 4C). Bray-Curtis and Jaccard beta diversity based on fungi was significantly different in clinical cohorts (Fig. 1F, Table S1D). Alpha diversity based on GO in combined kingdom was significantly increased in later stages of endometriosis (Fig. 3B, Table S1D) while clinical scoring did not contribute to the significantly higher alpha diversity based on bacteria in microbiome group 4 (Fig. S2). Additionally, separating inflammatory controls added more diversity in separations (Table S1B), and controls with inflammation resulting in higher alpha diversity than non-inflamed controls, although this was not statistically significant (Fig. 3B).

Functional analyses of the microbiota indicate specific pathways and genes associated with changes in the microbiota composition both in endometriosis and other inflammatory gynaecological conditions (Table S1B). While the bacteria identified specifically in endometriosis patients were of low abundance, the increased alpha diversity suggested a functional microbiota change in the vaginal environment which may impact the host and exacerbate patient symptoms (Table S1B).

### Urine *N*-glycome in endometriosis

Serum glycans specific for endometriosis were identified previously from this cohort in addition to another separately collected cohort in a study looking for potential non-invasive serum biomarkers^17^. Here, we also investigated urine *N*-glycome from all glycoproteins, and IgG specifically, using sensitive UPLC-based profiling with Procainamide (ProA) labelling^22^. This has not previously been reported in endometriosis. 181 *N*-glycans from all glycoproteins were characterized in detail in the pooled human urine samples (Tables S2, S3). Main glycans and derived features are summarized in Table S5. The main group of glycans identified from both control and endometriosis pooled samples were complex glycans. The glycans were highly branched, over two thirds were galactosylated and more than half of the glycans were sialylated. About half of the glycans from the samples were also fucosylated; however, the composition differed (Table S3). Glycans from the endometriosis-associated pool encompassed more complex glycans with a decrease in oligomannose and hybrid glycans. The endometriosis pool had significantly increased branching, polylactosamine extensions, galactosylation and sialylation including presence of glycolylneuraminic acid. Fucosylation was also altered, with core fucosylation increased and outer arm fucosylation decreased. Changes in modified glycans were found: sulfation was increased and acetylation and the presence of GalNAc was decreased in the patient pool.

After correction for multiple testing, there was no significant change in endometriosis in the overall identified urine glycome when compared to that obtained from the control group.

### Urine and serum IgG *N*-glycome differ

ProA-labelled urinary IgG *N*-glycan composition was determined by HILIC-UPLC analysis and 55 distinct structures were assigned (Table S4A, B) and Pro-A labelled serum IgG was asssigned for comparison (Tables S4C, D, Table S5). To date, urinary IgG glycosylation has not been described in the literature. Several of the identified urine structures overlapped with those identified in serum^23^. This was expected because urine is a plasma filtrate. We identified four previously unrevealed modified *N-*glycans on IgG, namely, A1GalNAc1[SO_4_^−2^]1, FA1GalNAc[SO_4_^−2^]1, A2G1GalNAc1[SO_4_^-2^]1S1 and FA2G1GalNAc[SO_4_^−2^]1S1.

All percentage areas of each peak and derived features in each sample are provided in Table S6. Statistical analysis of urinary IgG glycans showed no significant difference at any of the glycan peaks or features in endometriosis compared with control samples. This can be explained by the low number of samples and the fact that the samples were collected in different menstrual cycle phases. Alternatively, urine IgG may not be impacted by endometriosis.

**Fig. 5:**
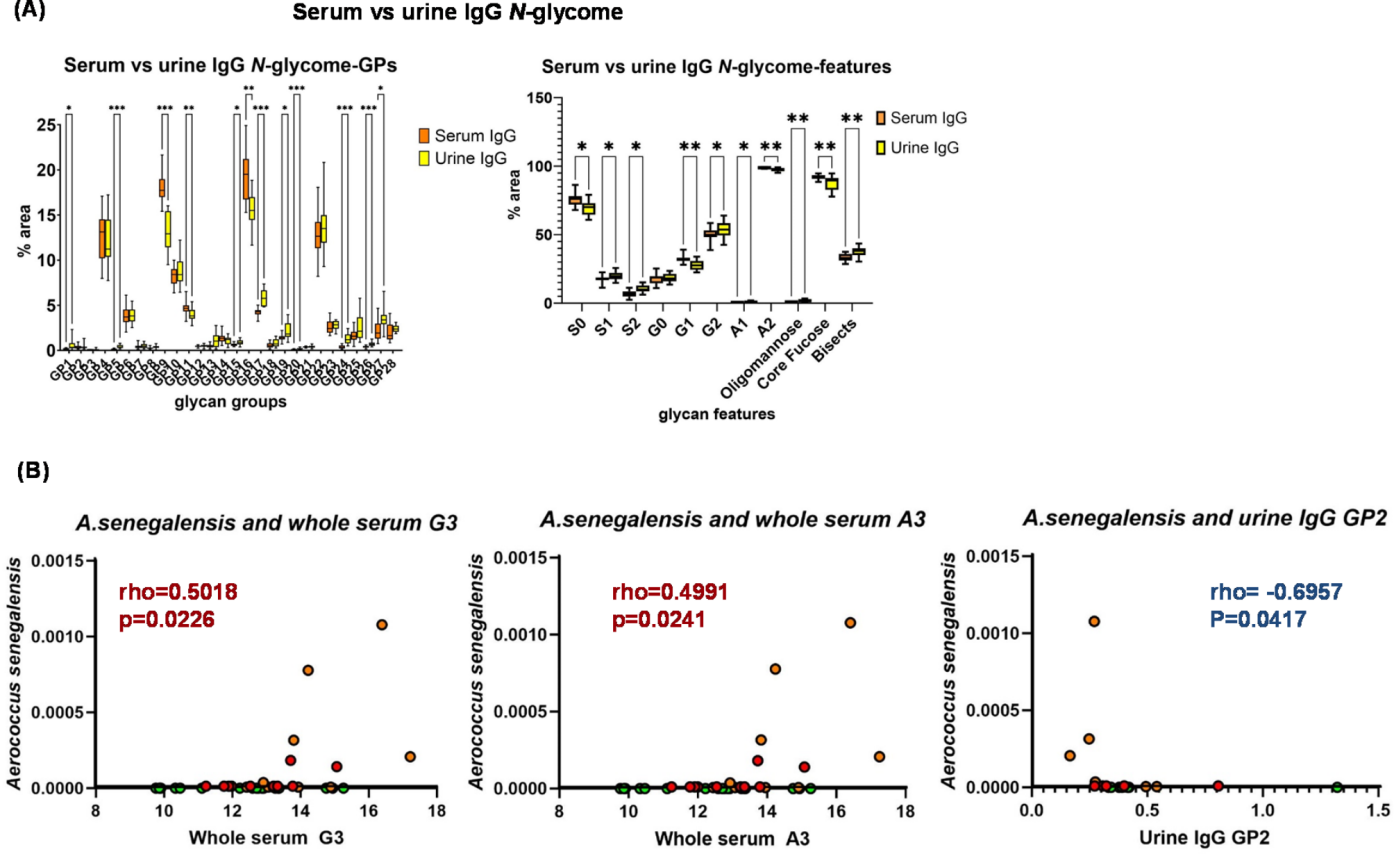
Glycosylation specific for urine and for endometriosis. (**A**) Glycome of urine and serum IgG differs and (**B**) specific glycans correlate with *Anaerococcus senegalensis*.

Next, urinary vs. serum IgG *N*-glycans were compared. In comparison to serum IgG, we found that most of the glycan peaks (GPs) were significantly different in the urine (Fig. 5A, Table S5D) both in control and endometriosis patients. Glycan features were also significantly different, namely increases in sialylation, galactosylation, oligomannosylation and bisecting glycans, and decreases in branching, and core fucosylation in urine IgG in general (Fig. 5A, Table S5D).

### Microbiota link to clinical features

All data were correlated in order to assess the relationship among microbiota, glycans and clinical features in endometriosis (Table S7). Individual *N*-glycan GPs and features were corrected for multiple testing.

**Fig. 6:**
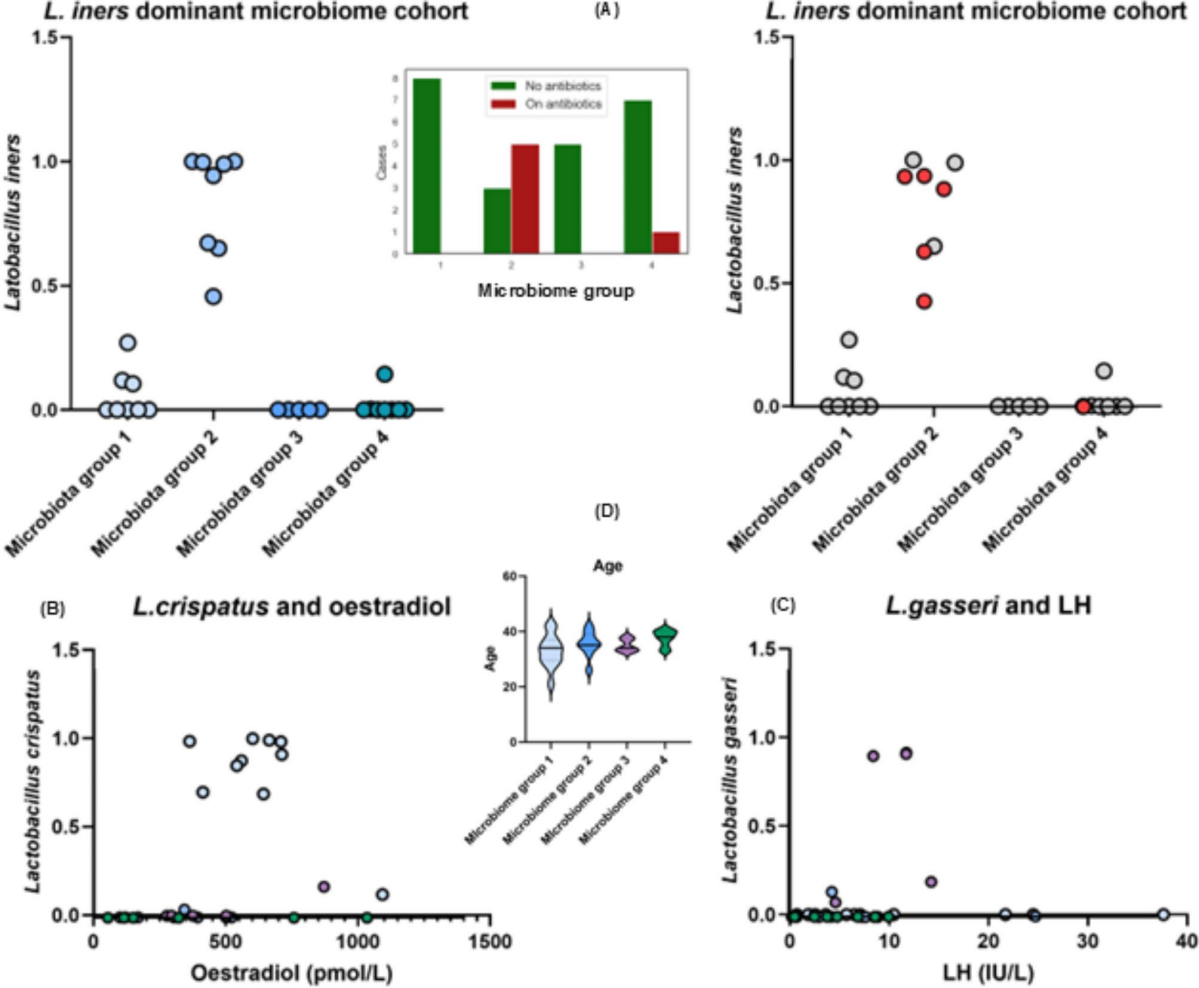
Microbiota group correlations with clinical characteristics. (**A**) *Lactobacillus iners* group (microbiota group 2) has most users with antibiotics (*Lactobacillus iners* correlate with antibiotic use, red colour indicates antibiotic users). (**B**) *Lactobacillus crispatus* group (microbiota group 1) correlates with oestradiol levels. (**C**) *Lactobacillus gasseri* group (microbiota group 3) correlates with LH levels. (**D**) Microbiota group 4 is slightly older.

Table S8A, B outlines the most significant correlations between clinical diagnosis of endometriosis, other clinical factors and microbiota. Several bacteria were shown to correlate with endometriosis clinical groups such as *Alloscardovia_u_s*,* Ureaplasma urealyticum*,* Anaerococcus obesiensis*,* Parvimons_u_s*,* Prevotella melaninogenica*,* Gardnerella_u_s*,* Peptoniphilus vaginalis* and *Anaerococcus hydrogenalis*. Other microbes enriched in endometriosis were *Candida albicans* (a fungal species) and a *Streptococcus phage* phiARI0746 (Table S8A). The bacterial species *Anaerococcus senegalensis*,* Prevotella jejuni*,* Porphyromonas bennonis* and *Anaerococcus octavius* were found to be specific for endometriosis occurring in more than 3 patients, see Table S1B, C and S8A. Interestingly, many of endometriosis-specific microbes were associated with typical clinical factors important for endometriosis, mainly peritoneal endometriosis and the presence of endometriomas, which also correlate with clinical cohorts (Table S8A, B). Clinical cohorts correlate with other inflammatory gynaecological conditions (mainly in controls), LH and age (controls were slightly older, Table 1). Peritoneal endometriosis correlates with *Anaerococcus senegalensis* which is associated with endometriosis. Endometrioma correlates with diagnosis of endometriosis and many microbes associated with endometriosis, mainly *Prevotella jejuni*. Endometrioma also affects menstrual cycle length. Such cycle length associates with FSH and oestradiol as well as with some microbes. Phase of the menstrual cycle associates with hormones, mainly progesterone. Antibiotics are well known to significantly affect microbiota composition in female reproductive tract^24^, while antibiotics also correlate with cycle phase and insulin as these may be affected by microbiota changes. Inflammatory gynaecological conditions do not correlate with endometriosis-specific microbes. Separation into microbiota cohorts could be driven by various clinical factors, e.g. *Lactobacillus iners* correlates with antibiotic use (most subjects using antibiotics had dominant *L.iners*), *Lactobacillus gasseri* correlates with LH and *Lactobacillus crispatus* with oestradiol levels (Fig. 6A-C). The *G.vaginalis* group (i.e. group 4) is slightly older than subjects belonging to the other microbiome groups (Fig. 6D).

### *N*-glycome links to specific microbiota

Endometriosis diagnosis does not correlate with any glycans when adjusted for multiple testing. Interestingly, *N*-glycome correlates with the microbes specific for endometriosis *Anaerococcus senegalensis*, namely, core fucosylated mono-antennary glycans (GP2) from IgG and trigalactosylated and triantennary glycans from serum (Fig. 5B, Table S8C, D). Certain glycans also associate with other microbes that are not associated with endometriosis (Table S8C, D). The glycome also correlates with clinical factors associated with endometriosis diagnosis such as endometrioma, peritoneal endometriosis and age (Table S8C, D). Mainly urine glycans associate with diabetic markers such as fasting glucose and Hb1A1c (Table S8C, D).

## Discussion

There is a diversity of findings on vaginal microbiome in endometriosis in literature. The studies vary widely mainly due to heterogeneity of controls and patient populations, country, diagnostic criteria used and techniques of microbiota detection^12,25^. In addition, metadata outlining antibiotic usage, age, race/ethnicity, menopausal status and timing of sample collection in relation to diagnosis of endometriosis was not consistently reported^26^. Most studies found changes in vaginal microbiome in endometriosis except for Perrotta et al.^27^. This study was based on 16 S rRNA gene-based amplicon sequencing methodology, which is less sensitive than the metagenomic approach used in this study^12,27^.

We have described the vaginal microbiome of endometriosis patients and their unique glycome (serum and urine) in comparison to a cohort of other gynaecological patients. By employing whole genome sequencing, we have identified species-specific microbial populations of the vaginal microbiome, such as specific *Lactobacillus-*or other microbiota-dominant populations. We also identified that disruption of the dominant *Lactobacillus* population results in increased diversity of the microbial populations, with known pathogens such as *Gardneralla*,* Prevotella*,* Ureaplasma*,* Anaerococci*,* Alloscardovia* and *Candida*. We found novel bacteria (*Parvimonas* and *Facklamia*) and a *Streptococcus phage* phiARI0746 not previously explored in endometriosis. *Gardnerella vaginalis* is associated with dysbiosis^28^, and this links to the increased alpha diversity, demonstrating the loss of keystone species such as certain species of *Lactobacillus*, suggesting vaginal microbiota imbalance. Particular *Lactobacillus* species are presumed to be important in sustaining a healthy vaginal microenvironment and a decrease of these species could have negative effects on reproduction^29–31^, as noticed in our cohort of subjects attending a fertility clinic. From a host viral perspective, five subjects had detectable Human endogenous retrovirus K (HERV-K, two controls, two stage 1 endometriosis and one stage 3 endometriosis patients), being mainly associated with microbiome groups 3 and 4. HERV-K is a human retrovirus which is associated with cancer and sclerosis^32^. One control had detectable levels of Alphapapillomavirus 6, at low risk of development of cervical cancer^33^.

Muraoka et al. found significantly more *Fusobacterium*, in particular *F. nucleatum*, in endometrial tissues and also in vaginal swabs from patients with endometriosis compared to controls without endometriosis^5^. In our study, *Fusobacteria* were found in two laparoscopy-confirmed controls and one patient with advanced endometriosis (*F. gonidiaformans* and *F. nucleatum* were found in one control and one patient, and *F. equinum* in one control) (Table S1A), indicating that the presence of *Fusobacterium* species is not specific for endometriosis. Although our controls represent non-healthy individuals, similar to the Muraoka at al. study^5^, this finding may depend on the particular samples and detection methodology selected for the study (16 S rRNA versus shotgun metagenomics). Perrotta et al. found that an increase in members of the genus *Anaerococcus* (specifically *Anaerococcus lactolyticus* and *Anaerococcus degenerii*) predicts endometriosis severity^1,27^. While *Anaerococcus* was also found enriched in endometriosis in our study, *A. lactolyticus* was not specific for endometriosis as it was also found in controls and *A. degenerii* was not found in our study. *A. octavius* and *A. senegalensis* were both more specific for mild/minimal stages of endometriosis rather than moderate/severe disease (Table S1B, Figs. 3A, 4A). It has been suggested that higher proportions of strictly anaerobic bacteria (including *Prevotella*,* Anaerococcus*), are linked with inflammation or dysbiosis in the vagina^10^. *Prevotella jejuni* was increased in endometriosis vaginal samples and increased with disease progression^10,12^ in agreement with our study (Fig. 3A, 4A , Table S1B). There are also studies that reported a decrease in *Prevotella*, but this may reflect genus specific studies rather than species or strain specific analyses^9,12^. Vaginal *Porphyromonas* spp. were found to have potential to alter the homeostasis of reproductive tissues and harm human pregnancy through clotting disruption, foetal membrane weakening, and premature cervical remodeling^34^. Prevalence of the *Gardnerella vaginalis + Prevotella bivia + Porphyromonas spp.* group in endometrium was associated with a low abundance of *Lactobacillus spp*. in infertile patients including individuals suffering from endometriosis^35^. *Alloscardovia*^11,12^. *Ureaplasma*^1,9,10,12^, *Anaerococcus*^12^, *Veillonella*^10–12^, *Corynebacterium*^10^, *Gardnerella*^1,9–11^, *Peptoniphilus*^10^, *Candida albicans*^36^ were found to be associated with endometriosis. Many of these bacteria were found to be associated with vaginal community state type linked with vaginal inflammation or dysbiosis^10^. The relative abundance of the genus *Lactobacillus* has been found to be decreased in endometriosis, including vaginal samples^10^, in agreement with our study (Table S1B, Figs. 3A and 4C). Indeed, all these microbial genera (and species) were detected in our sample cohort. *Parvimonas* and *Facklamia* have not yet been linked to endometriosis. Antimicrobial resistance genes, virulence factors and phages have not yet been explored in endometriosis. Interestingly, a larger abundance of phages was found in some inflammatory diseases, which may link to disease pathogenesis^37^. Endometriosis was found to be associated with higher alpha diversity in the vagina and changes in beta diversity^38^, in agreement with our results. One endometriosis patient with advanced stage 4 disease was found to have a dominance of *Streptococcus* sp HMSC034A12 and was 40 years old. This is not surprising, as the presence of vaginal *Streptococcus* is associated with post-menopausal status and this patient was on a GnRH analogue at the time of her surgery so this would have rendered her temporarily menopausal^39^.

Apart from found dysbiosis, low abundant, intracellular pathogenic microbes, which have potential to affect a local environment sensitive to dysbiosis^4^, such as *Ureaplasma* or *Fusobacterium*, were found in endometriosis^1,5,9^. *Fusobacterium nucleatum* has been found to influence the endometrial environment and promote endometriosis^5^. Antibiotics have been shown to reduce the endometriotic lesions in mice and to improve pregnancy rates in humans^5,24^, highlighting the importance of microbiota in endometriosis. However, the consequences of an altered microbiome in endometriosis patients have not yet been fully established, nor is it known why only some women develop endometriosis and related infertility. A host-derived factor that may play a role in the aetiology of endometriosis is altered glycosylation of host proteins interacting with pathogenic microbes.

Urine constitutes a challenging analyte in the field of glycomics. Variations of the normal urinary proteome^40^ and variations in the adult as compared to the paediatric urine glycome^41^ were reported. Furthermore, a glycopeptide study indicated that IgG *N*-glycosylation is associated with inflammation status in chronic kidney disease and this has the potential to be a novel biomarker for kidney diseases^42^. The detailed urine *N*-glycome characterization was not reported before in endometriosis. Using a combination of HILIC-UPLC, LC-MS and exoglycosidase digestions, we identified 181 *N*-glycans in the human urine samples and 55 distinct structures on urine IgG. To date, urinary IgG glycosylation has not been described in literature. We have described four previously unrevealed modified *N*-glycans on IgG. Analyses of pooled samples from endometriosis and controls revealed differences in glycosylation of urine glycoproteins in endometriosis. Sialylation has previously been found to be dysregulated both in endometriosis patient serum and endometriotic tissues^1^. An increase in sialylation indicates ongoing inflammatory processes. The increase in core fucosylation in endometriosis may reflect high levels of oestrogen associated with endometriosis, as an association between oral contraceptives and hormone therapy has been observed^43^. The decrease in outer arm fucosylation is unexpected, as inflammation is usually associated with an increase in these glycans^14^. Core fucosylated and bisecting glycans are decreased with age^14^. Altered levels of acetylated, sulfated glycans and glycans containing GalNAc may also play an immune role in endometriosis. Correct glycosylation is important for implantation and these changes may not only reflect an altered immune response but may also be responsible for implantation failure and infertility in endometriosis.

IgG is a well-studied glycoprotein and it is well-known that its immunological properties are modulated by its glycosylation^44^. Comparison of urinary vs. serum IgG *N*-glycans revealed significantly different glycosylation, namely increases in sialylation, galactosylation, oligomannosylated and bisecting glycans and decreases in branching, and core fucosylation in urine IgG in endometriosis, reflecting the distinct immune function of the urinary tract. The permeability and selectivity of the kidney vary with respect to the size and charge of the filtered plasma proteins^45^. It is already known that proteins in the urine receive additional posttranslational modifications (PTMs) that modify their properties^46^ and that the non-enzymatic glycosylation and additional PTMs of the IgG significantly increase its vascular clearance rate, which can explain the observed modified structures on IgG^47^. Therefore, it was not unexpected that urinary *N*-glycans also contained PTMs, possibly to help protein excretion. IgG sialylation on the Fc region decreases its affinity to the Fcγ receptor, which was proven to be anti-inflammatory^48^. Increased sialylation increases a protein’s half-life, while increased oligomannosylation has the opposite effect through increased antibody clearance^48^. Decreased core fucose and increased bisects indicate enhanced antibody-dependent cellular toxicity (ADCC)^48^ of urine IgG in comparison to serum IgG. This indicates different immune functions in urine IgG in comparison with serum IgG.

We have explored the interconnectivity of microbiota, glycome and clinical features. Many endometriosis-specific microbes and glycans were associated with typical clinical factors important for endometriosis, mainly peritoneal endometriosis and the presence of endometriomas.

Dysbiotic microbiota affect oestradiol metabolism by increasing the circulating oestrogen in the body, resulting in a hyper-estrogenic state, known to contribute to the development of endometriosis^49^. Dominance of *Lactobacillus iners*^39^ is associated with post menopause. Although our cohorts represent premenopausal women, one would assume this specific microbiota increases with approaching menopause, however, *L. iners*-dominated group 2 is not significantly older than other subjects (Fig. 6D). Interestingly, one urine IgG *N*-glycan and two serum *N*-glycan features correlated with the presence of *Anaerococcus senegalensis*, identified in endometriosis vaginal swabs. The glycome and microbiota in endometriosis have not to date been linked, however, host glycans, such as blood group antigens and other glycans expressed on mucosal surfaces can serve as a receptors for microbes, affect susceptibility to infections and are linked to autoimmune pathogenesis^20,21^. The glycans therefore could be important mediators in endometriosis initiation, characterisation and maintenance. Urine glycans also associate with diabetic markers (such as fasting glucose and Hb1A1c), a finding that was previously unpublished, possibly due to under-exploration of the urine glycome.

### Clinical implications

 These observations highlight that endometriosis is a both a local vaginal and a systemic disease^11^, and contribute to understanding the pathogenesis and clinical manifestation of the condition, including hormone dysregulation, infertility, and a chronic inflammatory phenotype. The observed relationships between glycans, microbes and clinical features point to the potential causative role of low abundant pathogenic microbes. This was previously hypothesized^4^ and confirmed on *Fusobacterium nucleatum*^5^, “blooming” in a dysregulated vaginal environment where the dominant *Lactobacillus* species has been reduced with a host glycome representing a potential patient susceptibility factor.

### Strengths and limitations

Strengths of this study include the fact that we compared endometriosis cases with other gynaecological patients with similar symptoms, rather than healthy controls and measured large diverse potential biomarkers from multidisciplinary fields of glycobiology, microbiology and clinical features associated with endometriosis. While these results would need to be confirmed in larger and diverse patient populations, with serial sampling of urine, serum, swabs and endometrial lesions, at different timepoints to incorporate hormonal changes during the menstrual cycle, this study outlines the importance of system biology in the evaluation of disease aetiology. If further functional studies with a multidisciplinary research approach, prove causation by the described microbial imbalance, or by its sequelae we may well eventually identify the aetiology of endometriosis.

## Methods

### Study design

The Research Ethics Committee of the National Maternity Hospital, Dublin (Ref. No.:EC19.2018) approved this protocol. Written informed consent was obtained from all participants. This research was performed in accordance with the Declaration of Helsinki. All patients underwent laparoscopy for the investigation of infertility, pelvic pain or pelvic pathology suspected on ultrasound scan. Patients were separated into controls (19), minimal/mild (11) and moderate/severe (10) endometriosis. The severity of endometriosis was classified according to the revised American Society for Reproductive Medicine classification^50^. None of the women had been using any hormonal medications and/or anti-inflammatory therapy for at least three months prior to surgery. All women had negative screening results for sexually transmitted diseases, active systemic infection, a history of autoimmune diseases, active vaginosis; acquired or primary immunodeficiency diseases (including HIV), pregnancy, or a malignant condition. Inflammatory gynaecological conditions diagnosed by laparoscopy (hydrosalpinx, salpingitis, sactosalpinx), polycystic ovarian syndrome (PCOS), ovarian cysts, use of antibiotics and phase of the menstrual cycle were noted as well as the presence of an endometrioma or peritoneal endometriosis (Table S1A).

### Sample collection

**Peripheral blood samples** were collected using BD Vacutainer^®^ Venous Blood Collection tubes containing clotting activator (BD Company, Franklin Lakes, New Jersey, U.S.) before anaesthesia. Samples were processed within 1 h of collection. Serum was isolated from the peripheral whole blood samples by centrifugation at 2000×*g*/10min/4 °C. Separated serum samples were stored at − 80 °C until use.

**High vaginal swabs** were collected at laparoscopy. Some patients who did not have a vaginal swab taken at the time of laparoscopy, subsequently had this performed as an out-patient in the mid-luteal phase of the cycle. Samples were preserved in DNAgenotek.

**Urine samples** were obtained from participants in the morning (fasting) at the hospital prior to any treatment or surgery and stored – 80 °C before processing.

Additionally, pooled healthy human urine (Innovative Research, MI, US) was purchased for the development of techniques for the isolation and *N*-glycan profiling of whole urine glycoproteins and IgG protocols and for *N*-glycome identification of urinary IgG. Normal healthy serum purchased from Sigma Aldrich was used for serum IgG characterization.

### Hormone and glucose metabolism markers

Luteinizing hormone (LH), follicle stimulating hormone (FSH), progesterone, insulin and haemoglobin A1c (HbA1c) were measured using routine clinical lab tests. Whole blood was collected into lavender top vacutainers with ethylenediaminetetraacetic acid (EDTA) anticoagulant for HbA1c analysis. For fasting glucose, blood was collected into grey top tubes (with sodium fluoride, glycolytic inhibitor). Serum samples were used to measure LH, FSH, oestradiol, progesterone and insulin.

### Glycan analyses

Method development and reproducibility of the method was done using purchased normal human urine (Fig. S3).

### Pre-treatment of urine samples

Urine pre-treatment was performed using an Avanti J-E, High-Speed Centrifuge with JA-12 Fixed-Angle Aluminium Rotor (Beckman Coulter, CA, USA). Ten mL of urine samples were centrifuged at 15 min, 2000×*g* in a 50mL Falcon tube (MERCK, NJ, USA) to remove cell debris. Then the supernatant was centrifuged for 30 min, at 2000xg through a 10 kDa Macrosep Advance Centrifugal Devices (Pall Corporation, NY, USA). The filter was washed with ultra-pure water (ddH_2_O) at 15 min, 2000×*g* and the retained liquid was collected into a 2mL Eppendorf tube. One g/mL solution of trichloroacetic acid (dissolved in concentrated cold acetone) (Fisher Scientific, MA, USA) was added to the pre-concentrated urine samples in a 2:1 ratio. Samples were mixed and left to sit on ice for 45 min and then spun at 14,000×*g* for 5 min. The supernatant was discarded and 300 µL of cold concentrated acetone was added to the samples. Samples were mixed and spun again with the before-mentioned spinning conditions. The supernatant was discarded, and the pellets were used for *N*-glycome profiling of the whole urine samples.

### Pre-treatment for IgG glycan analysis

The above-described protocol was applied in the case of urinary IgG concentration, except that the TCA treatment was not applied. The retained liquid of the filter was transferred into 96-well plates. No special pre-treatment of serum samples was required before serum IgG capture and 50 µL serum was used to perform the below-detailed protocol. Both the urinary and the serum IgG were captured using Protein G PhyTips (PTH 91-20-02 Box of 96 PhyTip columns) and a Buffer kit of PhyNexus (BUF-91-40-01) (Biotage, Uppsala, Sweden). Pre-equilibrated Phytips were used for IgG capture (equilibration with Buffer A solution 200 µL per well, 20 cycles, at lowest speed; IgG capture with Buffer A solution and concentrated urine sample in 1:1 ratio, 30 cycles, at lowest speed). The captured IgG in the Phytip resin was washed with Wash buffer I (250 µL per well mixing volume, 10 cycles, at lowest speed) followed by a treatment of Wash buffer II (250 µL per well mixing volume, 10 cycles, at lowest speed). Then, IgG was eluted (250 µL per well mixing volume, 20 cycles, at lowest speed). Samples were neutralized with TRIS buffer, pH11.0 (MERCK, NJ, USA). All solutions used for the process were from the Buffer kit of PhyNexus except the 0.1 M citric acid reagent, pH2.5 used for elution (MERCK, NJ, USA), as this solution provided the best result after Peptide-*N*-Glycosidase F (PNGase F) treatment.

### *N-*glycan release

The whole serum and pre-treated urine samples were dissolved in 10µL of ddH_2_O and *N*-glycans were released using the high-throughput method described by Royle et al.^51^. Briefly, the samples were immobilized in SDS-gel blocks, reduced and alkylated in 96-well plates, and then washed. The *N-*glycans were released using peptide *N*-glycanase F (PNGase F, 50,000U/mL; NEB, cat. number P0709L), as previously described^52^. Neutralized IgG samples from urine and serum samples were centrifuged through a 10 kDa filter (Pall Corporation, NY, USA) at 10 min, 14000xg then 100uL of denaturation buffer (50mM dithiothreitol (DTT), 20mM NaHCO_3_, 0.1% SDS, MERCK, NJ, USA) was added to the filter and incubated at 20 min, 65 °C. After denaturation 40µL of 0.1 M iodoacetamide was added to each sample and incubated at 15 min, 40 °C (MERCK, NJ, USA). At the end of the incubation, samples were centrifuged at 10 min, 14000xg, then 100µL ddH_2_O was added to the samples at 10 min, 14,000×*g*. 20 µL PNGase F (1:400) in 20mM NaHCO_3_ buffer was added to each sample and incubated at 20 min, 37 °C to release *N-*glycans. After PNGase F treatment, the released glycans were centrifuged at 10 min, 14,000×*g* and collected into fresh Eppendorf tubes and dried in a SpeedVac system (Thermo Fisher Scientific, MA, USA).

### Labelling of glycans

Serum and urine *N*-glycans from all glycoproteins were fluorescently labelled with 2-Aminobenzamide (2-AB, MERCK, NJ, USA) by reductive amination^53^ and IgG *N*-glycans from urine and serum were labelled with ProA^22^. Excess 2AB and procainamide **(**ProA) dyes were removed on Whatman 3MM paper (Clifton, NJ, USA) in acetonitrile^51,54^.

### Exoglycosidase digestions

Enzymes were purchased from New England Biolabs (Hitchin, Herts, U.K.) or Prozyme (San Leandro, CA, USA). Either ProA or 2AB-labelled glycans were digested in a volume of 10µL for 18 h at 37 °C in 50mM sodium acetate buffer, pH 5.5 (except in the case of jack bean α-mannosidase (JBM) where the buffer was 50mM sodium acetate, 2mM Zn^2+^, pH5.0), using the panel of the following enzymes: sialidase cloned from *Streptococcus pneumoniae* and expressed in *E. coli* (NAN1, EC 3.2.1.18), 800 U/mL (NEB); sialidase cloned from *Arthrobacter ureafaciens* and expressed in *E. coli* (ABS, EC 3.2.1.18), 1000U/mL; β-galactosidase cloned from *Streptococcus pneumoniae* and expressed in *E. coli* (SPG, EC 3.2.1.23), 80 U/mL; β-galactosidase cloned from bovine testis and expressed in *Pichia pastoris* (BTG, EC 3.2.1.23), 200U/mL; α-fucosidase cloned from the sweet almond tree (*Prunus dulcis*) and expressed in *Pichia pastoris* (AMF, EC 3.2.1.111), 400 U/mL; α-fucosidase cloned from bovine kidney and expressed in *E. coli* (BKF, EC 3.2.1.51), 800U/mL; β-*N*-acetylglucosaminidase cloned from *Streptococcus pneumonia* and expressed in *E. coli* (GUH, EC 3.2.1.30), 8 U/mL (Prozyme) or 400U/mL (NEB); JBM (EC 3.2.1.24), 400 U/ mL (NEB). After incubation, enzymes were removed by filtration through a 10 kDa protein-binding filters (Pall Corporation, NY, USA)^51^.

### Liquid chromatography

Hydrophilic Interaction Liquid Chromatography–Ultra-Performance Liquid Chromatography (HILIC–UPLC) was performed using a BEH Glycan 1.7 μm and 130Å particles in 2.1 × 150 mm column (Waters, MA, US) on an Acquity UPLC (Waters, MA, US). Solvent A was 50mM formic acid adjusted to pH4.4 with ammonia solution and solvent B was acetonitrile. All urine glycoproteins: a 30 min method was used with a linear gradient of 30–47% with buffer A at 0.56mL/min flow rate for 23 min followed by 47–70% buffer A and reverting back to 30% buffer A. Urinary IgG samples: a 20 min method was applied with a linear gradient of 30–47% with buffer A at 0.56mL/min flow rate for 16 min followed by 47–70% buffer A and reverting to 30% buffer A. Samples were injected in 70% acetonitrile. 2-AB and Pro-labelled fluorescence was detected at 420 nm with excitation at 330 nm and at 370 nm with excitation at 310 nm, respectively. The system was calibrated using an external standard of hydrolysed and 2AB- or Pro-labelled glucose oligomers to create a dextran ladder^51^. The retention times of all identified peaks were given as glucose units (GU).

### Liquid chromatography–mass spectrometry (LC–MS)

Samples were cleaned up using Phynexus Phytip columns (PhyNexus, Inc, CA, USA) and separation was performed on a Q Exactive Plus (Thermo Fisher Scientific, MA, USA) mass spectrometer coupled with fluorescence detection of liquid chromatographic (LC-FLD-MS) system. Solvent A was 50mM ammonium formate adjusted to pH4.4 and solvent B was acetonitrile. A 40 min linear gradient with a flow rate of 0.15mL/min: 28% Solvent A for 1.0 min, increasing to 43% Solvent A over 30 min, increasing to 45% Solvent A over 1 min; returning to 28% Solvent A over 4 min, then equilibrating with 28% Solvent A for 4 min. A 1.7 μm Waters BEH Glycan column (1 × 150 mm) was used for sample separation the fluorescence detection excitation/emission wavelengths were λex = 310 nm and λem = 370 nm for ProA-labelled samples, while λex = 330 nm and λem = 420 in case of the 2AB-labelled samples. Mass spectrometry measurement of ProA-labelled IgG samples were performed in positive mode, spray voltage 3.40 kV, Capillary temperature 320 °C, Aux gas heater temperature 300 °C, Sheath and sweep gas flow rate 30 and 10 L/h respectively, Scan range 450–2500 m/z, Resolution 70,000. Mass spectrometry measurement of 2AB-labelled whole urine samples were performed in negative mode, spray voltage 3.40 kV, Xcalibur software (Thermo Fisher Scientific, MA, USA) GlycoWorkbench 2.0 software were used for MS data evaluation. The accurate glycan molecular weight addition of 2AB and Pro are 120.0688 and 219.1736, respectively.

### HILIC-UPLC data analyses

Glycan assignments were done using HILIC-UPLC in combination with exoglycosidase digestions and liquid-chromatography mass spectrometry (LC-MS), and glycan profiling using HILIC-UPLC.

**Fig. 7:**
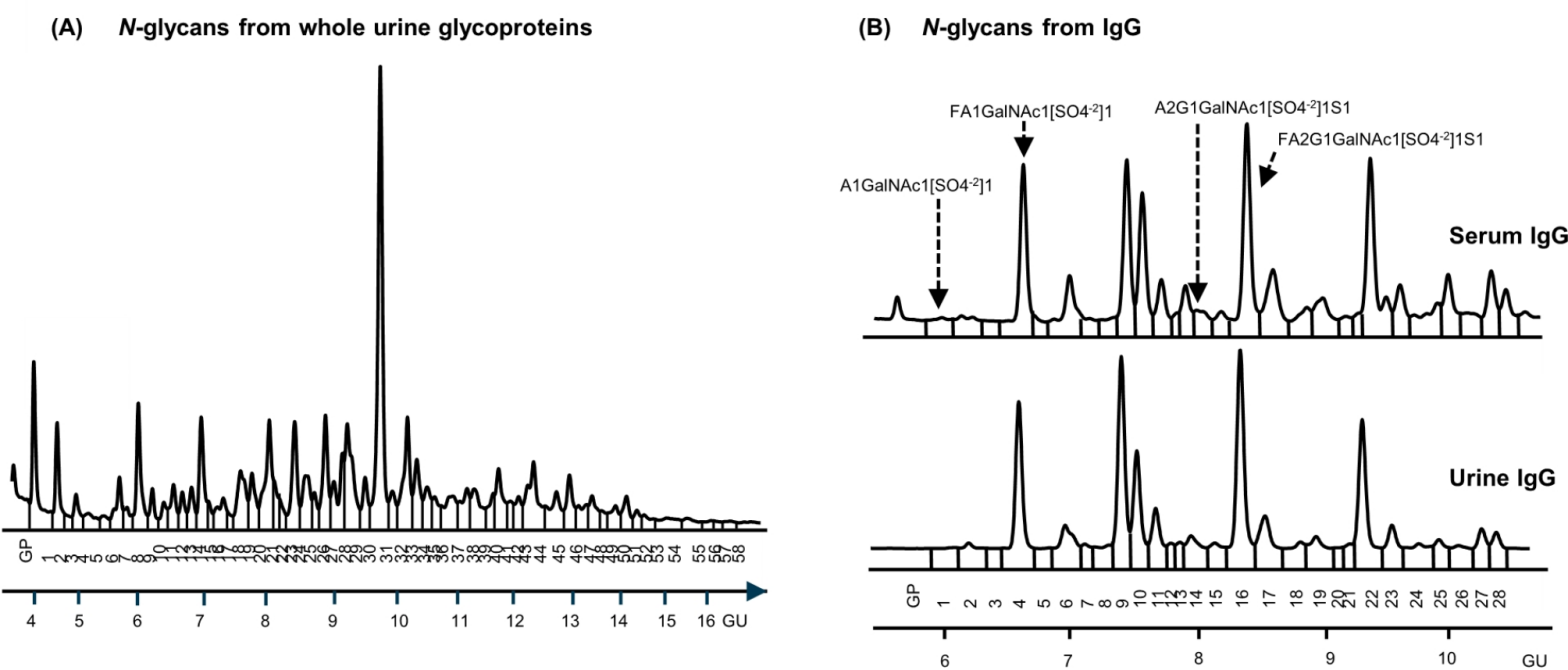
Representative HILIC-UPLC chromatograms of the *N*-glycans from. (**A**) All urine glycoproteins and (**B**) serum and urine IgG. Labelled are modified glycans found only in the urine IgG samples. Each glycan peak (GP) was numbered and detailed information about the assigned structures are presented in Tables S3 and S5.

Whole urine glycoproteins and IgG *N*-glycans run undigested on HILIC-UPLC were separated into 58 and 31 peaks, respectively. Coefficient of variations (CVs) in 58 peaks from whole glycoproteins and all 31 peaks from IgG *N*-glycans fell below 10% except for 2 small peaks (Fig.S3C). Overview of chromatograms and quantified glycan peaks (GPs) containing *N*-glycans from whole urine glycoproteins and IgG from urine from control and endometriosis patients is in Fig. 7A, B. Several representative samples were chosen to be pooled into these control and endometriosis pools. Sixty glycan peaks were integrated from the pooled control and 61 from the pooled endometriosis samples. The whole urine and IgG *N*-glycome from controls and endometriosis patients was separated on HILIC-UPLC into 58/31 well resolved GPs and the main glycans in these peaks were assigned based on characterized pooled samples (Fig. 7, Table S3-S5).

The chromatogram of pooled normal human urine IgG was integrated into 33 glycan and *N*-glycans assigned (Table S4A,B). Normal serum IgG was separated also into 33 peaks and 51 glycans were assigned (Table S4C,D) based on the publication of Pučić et al.^55^ but adjusted for the new method of ProA labelling.

Chromatograms of control and endometriosis urine IgG *N*-glycans were integrated into 31 glycan peaks (GPs). Chromatogram of *N*-glycans from control serum and urine IgG were re-allocated into 28 peaks to make consistent comparisons in samples where both sets of data were available (Table S5B,C).

### Glycan structure abbreviations

All *N*-glycans have two core *N*-acetylglucosamines (GlcNAcs); F at the start of the abbreviation indicates a core-fucose α1,6-linked to the inner GlcNAc; Mx, number (x) of mannose on core GlcNAcs; Ax, number of antenna (GlcNAc) on trimannosyl core; A2, biantennary with both GlcNAcs as β1,2-linked; A3, triantennary with a GlcNAc linked β1,2 to both mannose and the third GlcNAc linked β1,4 to the α1,3 linked mannose; A4, GlcNAcs linked as A3 with additional GlcNAc β1,6 linked to α1,6 mannose; B, bisecting GlcNAc linked β1,4 to β1,3 mannose; Gx, number (x) of β1,4 linked galactose on antenna; F(x), number (x) of fucose linked α1,3 to antenna GlcNAc; Sx, number (x) of sialic acids linked to galactose; Lac(x), number (x) of lactosamine (Galβ1-4GlcNAc) extensions.

### Glycan feature analysis

All glycans from serum and IgG proteins were categorized based on similarity in structure based on type (oligomannosylated, hybrid and complex), number of bisecting glycans (B), branching (mono-(A1), di-(A2), tri-(A3) and tetra-(A4)antennary glycans), based on fucosylation (F)(core fucosylated and outer arm fucosylated glycans), based on sialylation (neutral (S0), mono-(S1), di-(S2), tri-(S3), tetra-(S4) and polysialylated glycans), based on linkage of sialic acid (alpha2-3, alpha2-6 or both), based on galactosylation (non-galactosylated(G0), mono-(G1), di-(G2), tri-(G3) and tetra-(G4)galactosylated), modified glycans such as sulfated, acetylated, glycans containing polylactosamine extensions and GalNAc and glycolylneuraminic acid (Table S3). More details and explanation how these glycans were grouped is described and illustrated in Supplementary Tables S2, S3, and S5A,B.

### Microbiota analyses

#### DNA isolation and sequencing

DNA from high vaginal swab samples was isolated using an optimized protocol of varying size micro bead lysis accompanied by freezing at − 80 C was performed for efficient lysis overcoming sample connective tissue issues in addition to sample preparation in a laminar flow hood was performed^56^ using QIAamp UPC Pathogen Mini Kit (Qiagen) according to the manufacturer’s protocol. Whole genome sequencing (WGS) with functional analyses was performed (Table S1A).

Positive and negative extraction controls were incorporated into the extraction. Extracted DNA samples were quantified by qubit and microbial load verified by using 16srRNA qPCR and stored at − 80 °C until it was sequenced. Frozen samples were shipped on dry ice to the metagenomic services company CosmosID (Germantown, MD, USA). DNA samples were further quantified using the GloMax Plate Reader System (Promega) using the QuantiFluor^®^ dsDNA System (Promega) chemistry. The sequencing libraries were prepared by using the Illumina Nextera XT transposase system. DNA libraries were prepared using the Nextera XT DNA Library Preparation Kit (Illumina) and IDT Unique Dual Indexes were used. Genomic DNA was fragmented using a proportional amount of Illumina Nextera XT fragmentation enzyme. Unique dual indexes were added to each sample followed by 12 cycles of PCR to construct libraries. DNA libraries were purified using AMpure magnetic Beads (Beckman Coulter) and eluted in QIAGEN EB buffer. DNA libraries were quantified using Qubit 4 fluorometer and Qubit dsDNA HS Assay Kit. Libraries were then sequenced on Illumina NovaSeq platform following paired-end 150 bp sequencing protocol. Positive and negative controls and technical replicates were included for extraction and sequencing, all passed QC. The number of raw sequences reads per sample per species ranged within 36.87 M to 96.2 M (average 63.29 M per sample), control clinician glove samples revealed reads of 0.48 M reads matching to human and skin microbiota (Table S1A).

### Bioinformatic analysis

Unassembled sequencing reads were directly analysed using CosmosID-HUB Microbiome Platform (http://app.cosmosid.com, CosmosID Inc., Germantown, MD, United States).

The system utilizes a high-performance data-mining k-mer algorithm that rapidly disambiguates millions of short sequences reads into the discrete genomes engendering the particular sequences. The pipeline has two separable comparators: the first consists of a pre-computation phase for reference databases and the second is a per-sample computation. The input to the pre-computation phase are databases of reference genomes, virulence markers and antimicrobial resistance markers that are continuously curated by CosmosID scientists. The output of the pre-computational phase is a phylogeny tree of microbes, together with sets of variable length k-mer fingerprints (biomarkers) uniquely associated with distinct branches and leaves of the tree.

The second per-sample computational phase searches the hundreds of millions of short sequence reads, or alternatively contigs from draft de novo assemblies, against the fingerprint sets. This query enables the sensitive yet highly precise detection and taxonomic classification of microbial NGS reads. The resulting statistics are analysed to return the fine-grain taxonomic and relative abundance estimates for the microbial NGS datasets. To exclude false positive identifications the results are filtered using a filtering threshold derived based on internal statistical scores that are determined by analysing a large number of diverse metagenomes. The same approach is applied to enable the sensitive and accurate detection of genetic markers for virulence and for resistance to antibiotics.

### CosmosID-HUB functional analysis

Initial QC, adapter trimming and preprocessing of metagenomic sequencing reads are done using BBduk^57^. The quality-controlled reads are then subjected to a translated search against a comprehensive and non-redundant protein sequence database, UniRef 90. The UniRef90 database, provided by UniProt^58^, represents a clustering of all non-redundant protein sequences in UniProt, such that each sequence in a cluster aligns with 90% identity and 80% coverage of the longest sequence in the cluster. The mapping of metagenomic reads to gene sequences are weighted by mapping quality, coverage and gene sequence length to estimate community wide weighted gene family abundances as described by Franzosa et al.^59^. Gene families are then annotated to MetaCyc^60^ reactions (Metabolic Enzymes) to reconstruct and quantify MetaCyc^60^ metabolic pathways in the community as described by Franzosa et al.^59^. Furthermore, the UniRef_90 gene families are also regrouped to GO terms^61^ in order to get an overview of GO functions in the community. Lastly, to facilitate comparisons across multiple samples with different sequencing depths, the abundance values are normalized using Total-sum scaling (TSS) normalization to produce “Copies per million” (analogous to TPMs in RNA-Seq) units.

### Statistical analyses

Graphical, data and statistical analyses were performed using GraphPad Prism (version 10.0), online CosmosID software, Python 3 and associated Seaborn package. Variables were compared using non-parametric tests, namely, Spearman correlations, Mann Whitney or Kruskal Wallis with Dunn’s multiple comparison test. Relationship of microbiome groups with clinical factors and glycans were analysed using Kruskal Wallis or Chi-squared tests. Glycans were also corrected for multiple testing using Bonferroni (multiple GPs in one chromatographic profile) and paired data of serum and urine IgG were analysed using Wilcoxon matched-pairs signed rank test with Holm-Šidák method for multiple comparisons correction. The criterion for significance was set at *p value ≤ 0.05 or **p value ≤ 0.01 and ***p value ≤ 0.001, and ****p value ≤ 0.0001.

## Electronic supplementary material

Below is the link to the electronic supplementary material.

Supplementary Material 1

Supplementary Material 2

Supplementary Material 3

Supplementary Material 4

Supplementary Material 5

## Data Availability

Raw sequence data is available and has been deposited at NCBI SRA under the BioProject ID PRJNA1156592. Raw MS data for structural interpretation of *N*-glycans from control and patient samples are available on GlycoPOST (https://glycopost.glycosmos.org/)^61^ using the Project ID no. GPST000421. Python Jupyter notebooks and associated datasets are available at https://github.com/RadkaFahey/Endometriosis-urine-and-microbiome-paper.
