# Supplementary material for: Endometriosis specific vaginal microbiota links to urine and serum N-glycome
Source: Sci Rep. 2024 Oct 25;14:25372. doi: 10.1038/s41598-024-76125-2 (PMC11511964; doi:10.1038/s41598-024-76125-2)
Supplement: Supplementary file 1 — Supplementary Material 1 [file 41598_2024_76125_MOESM1_ESM.docx]

**ASSOCIATED CONTENT.**

**Supplementary Figures.**

**Supplementary Figure S1. Dominant microbiota groups with illustrated clinical cohorts**

(A) *Lactobacillus crispatus* dominant microbiota group 1

(B) *Lactobacillus iners* dominant microbiota group 2

(C) *Lactobacillus gasseri* dominant microbiota group 3

(D) Mainly *Gardnerella* *vaginalis* dominant microbiota group 4

**Supplementary Figure S2. Alpha diversities based on microbiota groups and bacteria with illustrated clinical cohorts.**

(A) Chao1

(B) Simpson

(C) Shannon

**Supplementary Figure S3. Method for urine *N*-glycome analyses and its reproducibility.**

(A) Schematic representation of whole urine and urinary IgG sample processing. Five mL of urine was concentrated with centrifugation then precipitated with TCA. Then the whole protein sample was diluted with 5 μL of ddH2O, denaturized, alkylated and treated with PNGase F enzyme. Finally, the released *N*-glycans were labelled with 2-AB, cleaned from excess dye and separated on HILIC-UPLC (i) . In the case of IgG capture, 10 mL of urine was concentrated, then the proteins washed with ddH_2_O and the remaining supernatant (500 μL) was used for IgG extraction and *N*-glycan release. For increased sensitivity, a fluorescent tag 4-amino-N-[2-(diethylamino)ethyl] benzamide (ProA) was used for labelling of the urinary IgG *N*-glycans at 65˚C, 1 hour, and the excess ProA was removed by using the above-mentioned clean-up protocol (ii).

(B) Reproducibility of whole urine *N*-glycome method

(C) Reproducibility of urine IgG *N*-glycome method

**Supplementary Tables.**

**Table S1A**. Clinical, microbiota and functional data. Bacteria are in relative abundance. In red are highlighted microbes above 1% relative abundance.

**Table S1B.** Microbiome and functional pathways specific for endometriosis and inflammatory controls.

**Table S1C**. Specific microbes for endometriosis patients and controls

**Table S1D.** Alpha and beta diversity in clinical cohorts

**Table S2A**. Exoglycosidase digestion panel for pooled control whole urine *N*-glycome

**Table S2B.** LC-MS data for pooled control whole urine *N*-glycome

**Table S2C.** Exoglycosidase digestion panel for pooled endometriosis whole urine *N*-glycome

**Table S2C.** LC-MS data for pooled endometriosis whole urine *N*-glycome

**Table S3.** Summary table of the assigned major *N*-glycan structures in each glycan peak and features of the whole urine glycoproteome from control (without endometriosis) and endometriosis sample.

**Table S4A.** Exoglycosidase digestion panel for control urine IgG *N*-glycome

**Table S4B.** LC-MS data for pooled control urine IgG *N*-glycome

**Table S4C**. Exoglycosidase digestion panel for control serum IgG *N*-glycome

**Table S4D**. LC-MS data for pooled control serum IgG *N*-glycome

**Table S5A**. Summary table of the assigned major *N*-glycan structures in each glycan peak of the urine and serum IgG *N*-glycan profile.

**Table S5B**. Features allocations for urine IgG *N*-glycome

**Table S5C**. Samples for comparison of urine and serum IgG

**Table S5D.** Changes in GPs and features in urine compared to serum IgG with p-values adjusted for multiple testing by Bonferroni. In red are increases and in blue decreases in urine compared to serum IgG.

**Table S6**. Clinical and glycome data

**Table S7A**. Correlations of clinical factors, microbiota and glycome- Spearman correlation (p-values)

**Table S7B.** Correlations of glycome with clinical factors and microbiota- glycome adjusted for multiple testing (Spearman and Bonferroni)

**Table S7C.** Correlations of clinical factors, microbiota and glycome- Spearman correlation (rho-values)

**Table S8A.** Relationship summary of clinical factors with microbiota

**Table S8B.** Summary of endometriosis associated microbiome correlations with clinical factors

**Table S8C.** Correlations summary of glycome with microbiota and clinical factor

**Table S8D.** Summary of glycans, clinical factors and microbiome
