# Supplementary figures and images for: Endometriosis specific vaginal microbiota links to urine and serum N-glycome
Source: Sci Rep. 2024 Oct 25;14:25372. doi: 10.1038/s41598-024-76125-2 (PMC11511964; doi:10.1038/s41598-024-76125-2)

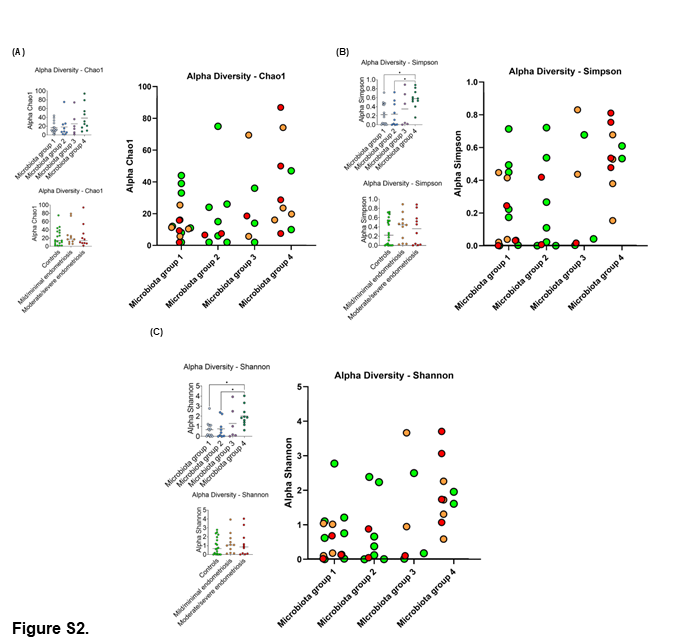

Supplement: Supplementary file 3 — Supplementary Material 3 [file 41598_2024_76125_MOESM3_ESM.tif]

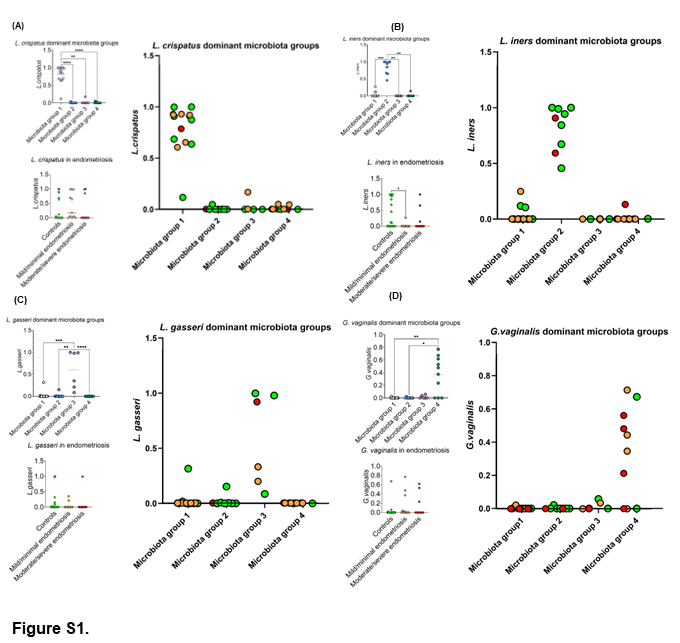

Supplement: Supplementary file 4 — Supplementary Material 4 [file 41598_2024_76125_MOESM4_ESM.tif]

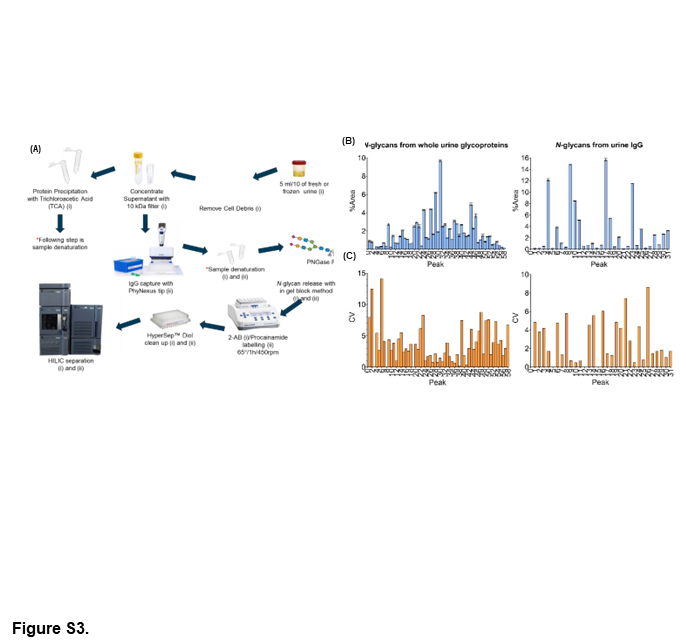

Supplement: Supplementary file 5 — Supplementary Material 5 [file 41598_2024_76125_MOESM5_ESM.tif]
